# Measurement of Physical Activity and Sedentary Behavior by Accelerometry Among a Nationwide Sample from the KiGGS and MoMo Study: Study Protocol

**DOI:** 10.2196/14370

**Published:** 2020-07-14

**Authors:** Alexander Burchartz, Kristin Manz, Bastian Anedda, Claudia Niessner, Doris Oriwol, Steffen CE Schmidt, Alexander Woll

**Affiliations:** 1 Institute for Sports and Sports Science Karlsruhe Institute of Technology Karlsruhe Germany; 2 Robert Koch Institute Berlin Germany

**Keywords:** processing criteria, wear time protocol, epoch length, sampling frequency, intensity classification, Motorik-Modul study

## Abstract

**Background:**

Currently, no nationwide objective physical activity data exists for children and adolescents living in Germany. The German Health Interview and Examination Survey for Children and Adolescents (KiGGS) and the Motorik-Modul study (MoMo) is a national cohort study that has incorporated accelerometers in its most recent data collection wave (wave 2, since 2014). This wave 2 marks the first nationwide collection of objective data on the physical activity of children and adolescents living in Germany.

**Objective:**

The purpose of this protocol is to describe the methods used in the KiGGS and MoMo study to capture the intensity, frequency, and duration of physical activity with accelerometers.

**Methods:**

Participants (N=11,003, aged 6 to 31 years) were instructed to wear an ActiGraph GT3X+ or wGT3X-BT accelerometer laterally on the right hip. Accelerometers were worn on consecutive days during waking hours, including at least 4 valid weekdays and 1 weekend day (wear time >8 hours) in the evaluation. A nonwear time protocol was also implemented.

**Results:**

Data collection was completed by October 2017. Data harmonization took place in 2018. The first accelerometer results from this wave were published in 2019, and detailed analyses are ready to be submitted in 2020.

**Conclusions:**

This study protocol provides an overview of technical details and basic choices when using accelerometers in large-scale epidemiological studies. At the same time, the restrictions imposed by the specified filters and the evaluation routines must be taken into account.

**International Registered Report Identifier (IRRID):**

DERR1-10.2196/14370

## Introduction

The health benefits of regularly performed physical activity are well documented in the public health literature. However, assessment of the physical activity of children remains difficult because the energy expenditure of a small active person can be as high as that of a large inactive person [1-4]. Because children show more complex but less structured movement behaviors than adults [3,5], capturing their many spontaneous and impulsive movements is a great challenge for physical activity assessment [6]. Currently, questionnaires are still the most commonly used subjective method to assess physical activity. One of the greatest advantages of questionnaires is their versatility. In addition to recording the duration, frequency, and intensity of physical activity, questionnaire methods can also be used to collect information about the type of physical activity, which has only recently become possible with accelerometers. Furthermore, in the context of large-scale epidemiological or health science studies, questionnaires are the only feasible alternative for practical and financial reasons [7]. In contrast, many empirical studies have already shown that the level of physical activity subjectively assessed by questionnaires is often overestimated [7-9]. Especially, the unstructured and irregular activities in everyday life are difficult to retrieve from memory correctly. In recent years, accelerometers have been used more frequently in large-scale studies [10-13] because they have become more feasible, more accurate, and much more affordable.

Although accelerometers are being used more frequently, there is no consensus on the usage of accelerometers for the assessment of physical activity in nationwide studies in adolescents or in children [14-16]. Due to the rapid development in this field and the extremely large amounts of gathered data, many current studies do not accurately document accelerometer use in detail (eg, technical details of settings and evaluation) [16]. This complicates replication and comparison of these studies because there are only a few representative studies worldwide [10,17]. Until 2014, no nationwide study had been performed in Germany in which physical activity was measured with accelerometers.

The aim of the Motorik-Modul study (MoMo), as part of the German Health Interview and Examination Survey for Children and Adolescents (KiGGS), was to establish regular monitoring of physical fitness and physical activity of children and adolescents living in Germany and to gain insight into their determinants and consequences for health outcomes. The MoMo study was established in 2003 and is based on a cohort-sequence design; four measurement waves (baseline and waves 1, 2, and 3) were planned from 2003 to 2021. Up to 2014 (baseline and wave 1), physical activity was assessed solely using a validated physical activity questionnaire (PAQ) [7]. In KiGGS and MoMo wave 2, physical activity was additionally assessed by accelerometers.

The purposes of this study protocol are to explain the challenges faced when using accelerometers in the MoMo and KiGGS studies as an example of a large-scale epidemiological study and to detail the methods and protocols used to capture physical activity in children and adolescents with accelerometers in Germany.

## Methods

### Study Design

KiGGS is part of the German health monitoring system established by the Robert Koch Institute. The KiGGS research topics are physical health, mental health, health-related behavior, health care, prevention, and social and environmental determinants. The study design and sampling procedure are described in detail elsewhere [18]. The core KiGGS survey is supplemented by the MoMo study, an in-depth study to assess the physical activity and motor performance of children and adolescents living in Germany that is being conducted by the Karlsruhe Institute of Technology. MoMo is being carried out with a subsample of KiGGS participants, as described in [19,20]. The KiGGS team established temporary study centers in 167 sample points all over Germany. Participants aged 0 to 17 years were randomly chosen from 167 registration offices and invited for interviews, physical examinations, and laboratory tests. At the study centers, KiGGS participants were asked if they were willing to participate in the MoMo study. If they consented, the interviews and physical examinations for the MoMo study took place approximately six to eight weeks later.

To date, three assessments have been conducted in KiGGS and MoMo: baseline (2003-2006; sample sizes: KiGGS n=17,641; ages: 0 to 17 years; MoMo, n=4528; ages: 4 to 17 years), the first follow-up (wave 1) between 2009 and 2012 (sample sizes: KiGGS, n=12,368; ages: 0 to 17 years; MoMo, n=5106; ages: 4 to 23 years), and the second follow-up (wave 2) between 2014 and 2017 (sample sizes: KiGGS, n=15,023; ages: 0 to 17 years; MoMo, n=5689; ages: 4 to 30 years). Wave 3 of the MoMo Study is currently underway (2018-2020; compare [19-22]). For the follow-up surveys (waves 1, 2, and 3), participants in the baseline survey were reinvited (longitudinal subjects). In addition, for cross-sectional analysis, a new sample of children aged 0 to 6 years was drawn in KiGGS wave 1, and in wave 2, a new sample of participants aged 0 to 17 years was drawn. For detailed sample sizes, see [19,20]. In KiGGS and MoMo wave 2 (2014-2017), accelerometry was used for the first time in this study to measure physical activity.

### Accelerometer Sample

**Figure 1:**
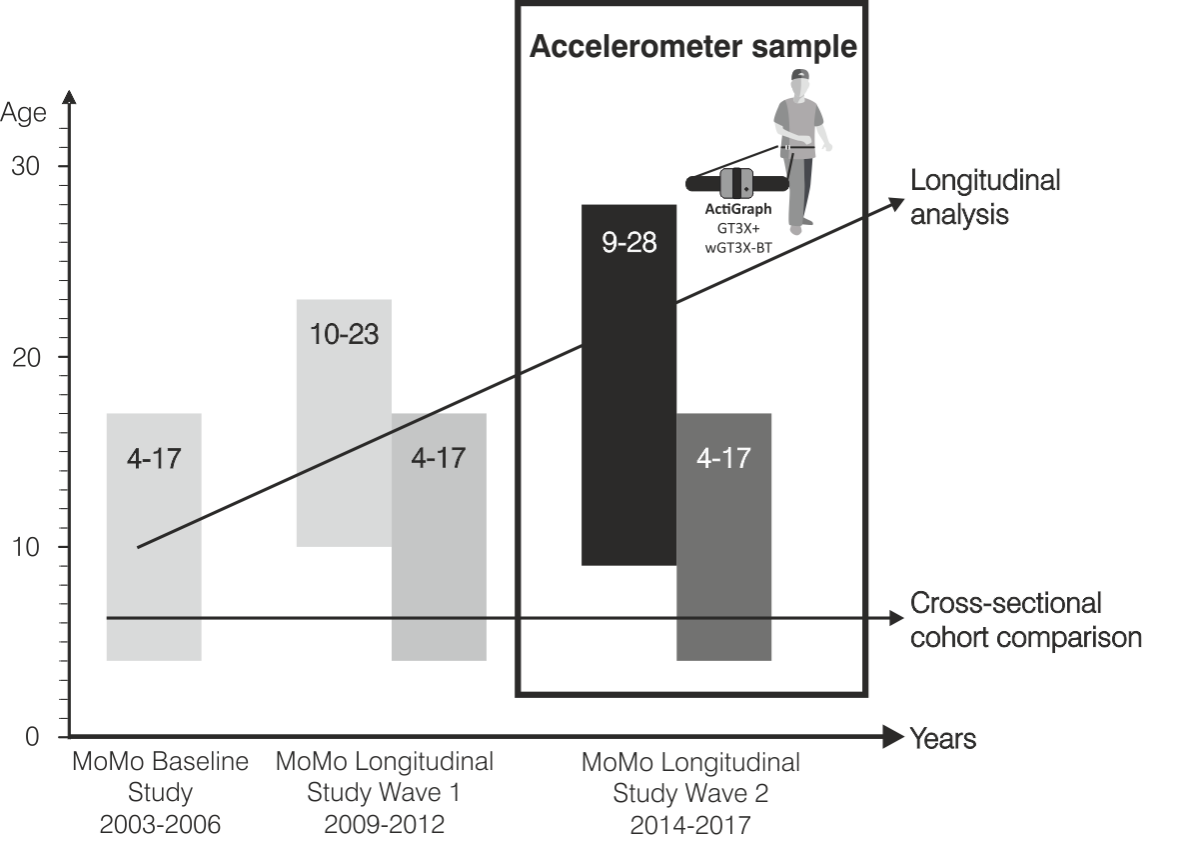
MoMo study design with the accelerometer sample in wave 2. MoMo: Motorik-Modul.

In KiGGS wave 2, all longitudinal participants aged ≥10 years (n=6465) were included in the accelerometer sample. In MoMo wave 2, all cross-sectional participants (n=4538) in the MoMo wave 2 sample who did not receive an accelerometer in KiGGS wave 2 were asked to wear one (Figure 1). Thus, a total of 11,003 participants were asked to wear an accelerometer. Participants who had impairments that prevented them from wearing an accelerometer were excluded. Participants who dropped out (did not agree to wear an accelerometer or experienced technical problems) in the MoMo and KiGGS studies are listed in Table 1 and Table 2, respectively.

**Table 1:** Details of the MoMo study participants asked to wear an accelerometer (N=4538), n (%).

Participation	Participants
Agreed to wear an accelerometer	2105 (46.4)
Dropped out because they did not agree to wear an accelerometer	2433 (53.6)
Downloaded accelerometer records	1974 (43.5)
Dropped out due to technical problems or did not wear the accelerometer	131 (2.9)

**Table 2:** Details of the KiGGS study participants asked to wear an accelerometer (N=6465), n (%).

Participation	Participants
Agreed to wear an accelerometer	5040 (78.0)
Dropped out because they did not agree to wear an accelerometer	1425 (22.0)
Downloaded accelerometer records	4750 (73.5)
Dropped out due to technical problems or did not wear the accelerometer	426 (6.6)

### Types of Accelerometers

In KiGGS and MoMo wave 2, ActiGraph accelerometers (models: GT3X+ and wGT3X-BT) were used to enable comparison with other large-scale European studies [10,13]. The heart rate monitor and Bluetooth wireless interface of each accelerometer were both deactivated during testing. The accelerometers were equipped with a tri-axial acceleration sensor (range: ±6g, sensitivity: 3mg, axes: horizontal right-left (x), vertical (y), and horizontal front-back (z)); they could record acceleration data at rates ranging from 30-100 Hertz and store it in epoch lengths from 1-240 seconds (compare with [23]). The settings are described in the “Initializing the Devices“ section. The physical dimensions of the devices were 4.6×3.3×1×5 centimeters, their weight was 19 grams, and they used a rechargeable lithium polymer battery.

### Assessment Period and Registration Protocol

MoMo and KiGGS accelerometer data sets were considered valid with a minimum wear time of 8 hours of recordings on 4 weekdays and 1 weekend day. These scoring policies are also consistent with the requirements for inclusion in the International Children’s Accelerometry Database (ICAD) [24]. Additionally, literature suggests that 4 days is a reasonable measurement time, thereby reducing the burden on participants and making it easier for researchers to collect sufficient data for the formation of recommendations related to general health guidelines [25-28]. To archive the highest possible number of valid data sets, the accelerometer should be worn for 7 days (8 days in the MoMo study) following the day of the examination in the study center. The assessment period of at least seven days ensured the inclusion of weekdays and weekend days. This inclusion is recommended due to differences in physical activity during the week and on weekends [25,29,30].

An additional analysis was planned of data sets with 10 or more hours of recording on each of the 5-7 days. This analysis was included because recent studies [30-33] propose the use of longer accelerometer wear times in hours and days to provide better estimates of daily activity.

The accelerometer should only be removed at bedtime, during activities that risk damaging the device (eg, martial arts), or when the participant is exposed to water (eg, swimming and showering).

### Initializing the Devices: Epoch Length, Sampling Frequency, and Filter

Each ActiGraph activity monitor was initialized using a standardized procedure prior to being given to the participant. The monitors used the latest firmware (v1.9.2. for wGT3X-BT and v3.2.1 for GT3X+), a unique output filename, and a sampling frequency of 30 Hz. In research with adults, the accelerometer signal was processed in epoch lengths of 10-60 seconds. Due to the sporadic activity of children, an epoch length between 1 and 5 seconds or the shortest possible epoch length is recommended [16,34-36]. The ActiGraph models used in the KiGGS and MoMo study store the collected raw data. Furthermore, the data are downloaded in epoch lengths of 1 second, reducing memory space and enabling faster data processing afterward. The devices can be used with two different filters when processing the data: a normal filter and a low-frequency extension filter (the implementation and algorithms of the filters are not open to the public). It is known that the normal filter detects accelerations in a range of 0.25-2.5 Hz [37]. To capture slower movements, the low-frequency extension filter establishes an unknown lower threshold [23]. While performing physical activity in a vigorous state, the human body produces accelerations at the hip up to 3.4 Hz [38,39]. Even higher frequencies were documented in the wrist when performing physical activity [40,41]. Considering these limitations, the normal filter was configured to recognize as many accelerations as possible.

In KiGGS, the device was set up to start measurement at 12:00 AM the day after the examination and to stop the measurement at midnight after 7 days of recording. A pilot study prior to data collection in MoMo revealed that many participants were confused by the standard “no flashing” mode of the ActiGraph device while recording. Therefore, “flash mode,” in which the device showed a green flashing light-emitting diode (LED), was activated during recording. In MoMo, the device was programmed to start at 12:00 AM on the day the participants underwent their motor performance tests to avoid the “no flashing” confusion noted above. The measurement stopped at midnight after 8 days of recording. In the MoMo study, the recordings of the first day were not considered for data analysis because the participants received the devices at different times throughout the day depending on the initial timing of their examination. Additionally, the first day served as an adaptation period for the participants.

### Placement of the Device

**Figure 2:**
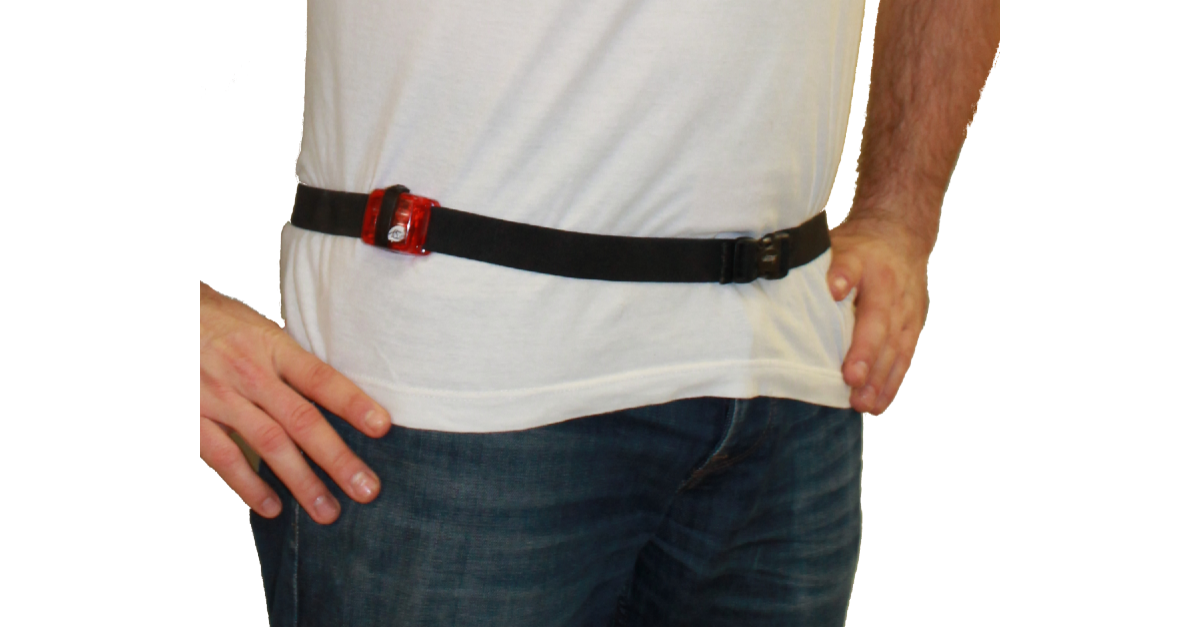
Participant wearing an accelerometer device on top of the right anterior superior iliac spine.

In KiGGS and MoMo, the device was placed laterally on top of the right anterior superior iliac spine with the closure on top, then secured with an elastic belt (see Figure 2). Compared to wrist attachment of the device, the hip monitor placement provides better acceleration detection due to the limited frequency range of the normal ActiGraph filter. The higher movement frequencies at the wrist would be out of the range of that filter. Moreover, most cut points for intensity estimation are validated with the device placed on the hip [16,41-43], and it is the most common carrying position for accelerometers [44]. More importantly, studies show more accurate classification of intensities when the device is placed on the hip than on the wrist [16,41,42,45,46].

### Nonwear Time Protocol (Logbook)

**Figure 3:**
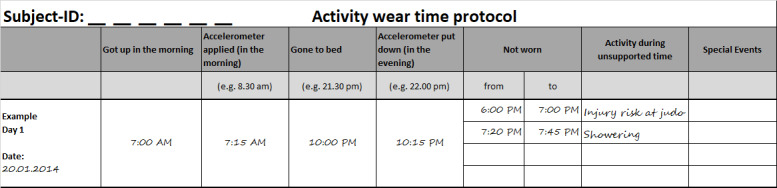
Sample MoMo wear-time-protocol (translated).

The accelerometer can only capture accelerations when it is worn; therefore, detailed information about the type of activity and the reasons for not wearing the devices is needed for complete understanding of the assessed physical activity. Therefore, participants were asked to complete a nonwear time protocol (see Figure 3). The combination of self-reports and device-based measures enables better understanding of physical activity behavior [47]. When both self-reported and device-based measured physical activity assessments are available, it is also possible to cross-validate the nonwear time calculated by the algorithms and the self-reported nonwear time. With information about the reasons for not wearing the device, statistics can be created for activities that were not captured and adjustment factors can be calculated.

### Transfer and Return of the Devices

Trained study assistants at the study centers distributed the devices. The participants chose the appropriate belt size and were shown how to wear the device correctly. Important aspects of wearing the accelerometer (placement, wear times, and return of the device) were summarized in an information sheet that was provided to the participants. At the end of the measurement period, the device, belt, and protocol were returned by mail. Therefore, an addressed and stamped envelope was provided to the participants. A follow-up protocol was implemented by telephone if the devices were not returned after 2 weeks.

### Data Download and Preparation

After receiving the devices, the data were downloaded as gt3x files using ActiLife Version 6.13.3 software (ActiGraph). The MoMo team marked all data sets with less than 4+1 days of wear time as invalid. In addition, ActiGraphData (AGD) files with data for all 3 axes and an epoch length of 1 second were created for data analysis. These data sets (1 second epoch length) can be converted into data sets with other epoch lengths, which enables comparison with studies using different data analysis protocols [42,43,48-52]. This is a faster way of processing the data than converting the raw data files (*.gt3x) to data files (*.agd) with different epoch lengths. Additionally, the time saved during the calculation is enormous when analyzing large data sets of several thousand participants. The resulting data sets would be the same. The gt3x files were stored separately to allow more in-depth analysis of the raw data after the planned evaluations (eg, with the GGIR software package [53]).

### Planned Data Processing

To ensure comparability with studies already included in the ICAD [24], we decided to conduct the analysis using the ActiGraph “count” system first. Before data analysis, data regarding nonwear time must be preprocessed. Therefore, the wear time values from the nonwear time protocols were compared with the calculated values of different nonwear time algorithms. The algorithms invented by Choi [51] and Troiano [52] were considered for the determination of wear time in this study. The Choi algorithm using a 90-minute window (±30 minutes) for capturing nonwear time was found to be the most practical because there is no need for 24-hour recording and the other nonwear time algorithms with a 60-minute window found too many incorrectly classified nonwear times [54]. Additionally, the nonwear time as identified by Choi is independent of the used epoch length [55].

Different cut points for physical activity intensity classification were calibrated for different epoch lengths. The most frequently used cut points were based on 1-second [42,48], on 15-second [43,49], or on 60-second [50,56] epochs. In our analysis, different cut points for intensity classification will be used for different age groups because the age range of the study sample includes children, adolescents, and adults (6-27 years). The cut points from Evenson [49] for participants aged 6 to 8 years, Hänggi [42] for participants aged 9 to 11 years, Romanzini [43] for participants aged 12 to 19 years, and Sasaki [48] for adult participants are currently under consideration. A summary of the accelerometer data processing criteria (suggested by Migueles et al [16], 2017) can be found in Table 3.

**Table 3:** List of accelerometer data processing criteria in KiGGS and MoMo (suggested by Migueles et al [16]).

Accelerometer data processing criterion	Definition in this study
Placement of the device	Laterally on top of the right anterior superior iliac spine
Sampling frequency	30 hertz
Filter	Normal ActiGraph GT3X filter
Epoch lengths	1 second with possibility to convert to 5, 10, 15, 30, and 60 seconds
Nonwear time definition	Choi et al 2011 [51]: 90-minute time window for consecutive zero/nonzero counts; allowance of 2-minute intervals of nonzero counts with an up/downstream 30-minute consecutive zero counts window
Valid days/valid weeks	8 hours of recording on at least four weekdays and one additional weekend day
Population age range	6-27 years (children, adolescents, and young adults)
**Sedentary and physical activity intensity classification and cut point algorithms^a^**	6-8 years: Evenson et al 2008 [49] 9-11 years: Hängii et al 2013 [42] 12-18 years: Romanzini et al 2014 [43] Young adults: Sasaki et al 2011 [48]

^a^To be determined; definitions listed are under consideration.

## Results

Data collection was completed in October 2017, and data harmonization was performed in 2018. The first accelerometer results from this wave were published in 2019 [57-59]. Detailed analyses are ready to be submitted in 2020.

First, data analysis should focus on gender and age differences of daily physical activity levels as well as compliance with the physical activity recommendations by the World Health Organization (WHO). Furthermore, we plan to perform in-depth analysis of the associations between physical activity and different health-related parameters (eg, obesity) and socioeconomic parameters (eg, education) by considering various data mining methods. This includes investigation of crosslinks and trends between questionnaires and device-based collected activity data (eg, physical activity differences between groups in both data sets).

## Discussion

### Summary

Currently, there are different concepts of collecting and processing accelerometer data for the assessment of physical activity among children and adolescents [16]. Many studies do not provide detailed descriptions of their data collection and data handling processes. This complicates replication of and comparability between studies. Therefore, the purposes of this study protocol were to explain the challenges faced when using accelerometers in the MoMo and KiGGS studies as an example of a large-scale epidemiological study and to detail the methods and protocols used to capture physical activity in children and adolescents with accelerometers in Germany.

### Strengths

This study protocol provides an extensive list of considerations for measuring physical activity and sedentary behavior by accelerometry in a large sample. These include technical details of the device used and the reasoning behind the device choice, the reasoning behind the a priori data collection proceedings (assessment period and registration protocol, device initialization, device placement, nonwear time protocol), and the data processing methods. Furthermore, thoughts on feasibility issues (transfer and return of the devices, data download and preparation) are provided. Researchers planning similar studies are given all the information needed for replication. This enables comparability to other large European studies such as the European Youth Heart Study [10] and the Healthy Lifestyle in Europe by Nutrition in Adolescence (HELENA) Study [13,36,60] due to similarities in methodology.

A multimodal approach of using self-reports and accelerometers is recommended [61] and can combine the advantages of the different methods (eg, precision and breadth of detection) because no single procedure provides optimal detection in all situations. The MoMo-PAQ was developed to measure habitual physical activity in general, while the current physical activity was measured by accelerometers over 1 week. Combining these two methods of assessing physical activity provides the opportunity to present a more comprehensive picture of the actual participant’s physical activity and can provide a basis for planning health-enhancing physical activity programs for specific target groups.

### Challenges

Although ActiGraph accelerometers are being used in many studies to record physical activity, there are technical issues associated with these devices; therefore, their limitations must be considered. The usage of the normal ActiGraph filter removes signals with a frequency greater than 2.5 Hz. However, while performing vigorous physical activity, the human body produces accelerations at the hip up to a frequency of 3.4 Hz [38,39]. Due to this limitation, activities with higher movement frequencies (ie, in the vigorous activity spectrum) may not be assessed correctly. In the context of MoMo and KiGGS, this will not be an issue because all activities in this frequency range will be classified as vigorous, and more detailed investigations are currently not planned. However, evaluating physical activity based on raw data is recommended for unbiased data processing that conforms to the open science approach. This requires more complex and advanced algorithms and evaluation methods. The first applied analysis will resort to a comparable evaluation with counts; however, future discussions on this topic are needed [62,63], and complex data analysis methods must be adapted.

Although unstructured and irregular everyday activities are recorded more accurately by accelerometers than by questionnaires, there are still improvements to be made. Devices can only measure physical activity when they are worn. Therefore, physical activity that occurs during nonwear time is not included in the data sets. This creates a need for methods that include additional information from these nonwear times, such as a nonwear time protocol that adjusts for the missing physical activity.

Taking into account the wide range of ages in the sample, different cut points and epoch lengths are suitable for the data in this study; however, calibration studies for such a broad sample do not exist. This leads to issues with accuracy of the data when only using one calibration study or to comparability issues within the sample when using multiple calibration studies for different subsets of the sample.

### Implications and Perspectives

For future waves of data collection, the nonwear time protocol should be improved. The frequency of reasons for nonwear must be analyzed so that the wearing instructions can be refined. This could potentially increase the wear time of the devices. Furthermore, the nonwear time protocol should assess the intensities of physical activity more precisely during nonwear times. This would lead to a more complete assessment of all occurring physical activity, and more detailed feedback could be given to the participants. Future studies should examine the accuracy of different algorithms for detecting nonwear times for different age groups [16]. The impact of different thresholds for physical activity intensity classification and of choosing the right epoch length for the target population based on age will be of interest as long as proprietary counts are used. Therefore, it is recommended to analyze multiple implemented cut point algorithms and identify the one that best fits the sample at hand.

Both methods of assessing physical activity should be compared between different target groups. Moreover, the adherence to physical activity recommendations by the WHO should be examined.

### Conclusion

This study protocol will help researchers obtain an overview of the decisions for the methods and protocols used to assess device-based physical activity in children and adolescents with accelerometers in Germany.

## Abbreviations

AGD: ActiGraphData
HELENA: Healthy Lifestyle in Europe by Nutrition in Adolescence
ICAD: International Children’s Accelerometry Database
KiGGS: German Health Interview and Examination Survey for Children and Adolescents
LED: light-emitting diode
MoMo: Motorik-Modul
PAQ: physical activity questionnaire
WHO: World Health Organization
